# Supplementary material for: Are carbon emissions trading and green financial instruments synergistic? -Comprehensive quantitative research based on content analysis
Source: PLoS One. 2024 Mar 7;19(3):e0298601. doi: 10.1371/journal.pone.0298601 (PMC10919601; doi:10.1371/journal.pone.0298601)
Supplement: S1 Data — (ZIP) [file pone.0298601.s001.zip › Data/地级市Z_F工作报告.zip!/╔┬╬≈/║║╓╨╩╨/└┤╫╘╣1⁄2╓┌║┼í╛┬φ┐╦╩2╛▌═°í┐_2017.docx]

各位代表：

　　现在，我代表市人民政府向大会作工作报告，请予审议，并请市政协委员和其他列席人员提出意见。

　　一、过去六年工作回顾

　　四届人大一次会议以来，在省委、省政府和市委的坚强领导下，全市上下围绕建设陕甘川毗邻地区经济强市、文化名市、宜居富裕城市目标，紧扣绿色循环、生态宜居战略定位，迎难而上、苦干实干，统筹推进稳增长、调结构、促改革、惠民生、防风险工作，圆满完成经济社会发展的各项目标任务，实现了“十二五”圆满收官、“十三五”良好开局。

　　━━综合实力整体跃升。六年来，主要经济指标位于毗邻地区前列，增速稳居全省第一方阵。生产总值年均增长12.2%，2016年达到1156.49亿元，总量由全省第7进到第6位；规模以上工业总产值年均增长19.2%，2016年达到1145.07亿元；财政总收入年均增长15.2%、2016年达到105.67亿元，地方财政收入年均增长15.9%、2016年达到45.19亿元。

　　━━产业结构不断优化。三次产业结构为17.8:42.8:39.4，一产占比较2010年下降3.9个百分点，二产、三产占比分别上升3.7、0.2个百分点。猪、药、茶、菜、渔五大产业产值占农业总产值比重达61.7%，主导作用更为明显；装备制造、有色冶金、绿色食药产业产值占规模以上工业总产值比重达77.9%，骨干支撑更为有力；旅游总收入相当于第三产业增加值的38%，对服务业的拉动作用尤为突出。非公经济占比达51.5%。

　　━━投资拉动成效明显。“十二五”期间，全社会固定资产投资完成3509.9亿元，是“十一五”的3.8倍。2016年完成1209.89亿元，增长24.5%、居全省第1位；民间投资增速达35.6%，居全省第1位；我市促投资稳增长的典型经验受到中省肯定。在投资的强力拉动下，一大批事关汉中长远发展的重大项目相继建成投用，城乡面貌焕然一新，发展支撑日益强劲。

　　━━深化改革蹄疾步稳。市级部门行政审批事项由520项减少到197项，精简率62.1%。率先在全省完成不动产登记制度改革，投融资体制改革创造“兴汉新区PPP典范”，教育体制改革和商事制度改革受到中省肯定，连续三年荣获全省医改先进市，组建了文化传媒集团。

　　━━生态文明成果丰硕。汉江流域污染防治三年行动取得显著成效，空气、水、土、声环境质量持续向好，年度减排任务均超额完成，中心城区空气质量优级天数居全省前列。森林覆盖率达59.1%，高于全省16个百分点。

　　━━人民生活显著改善。民生支出六年累计达1104.2亿元，占财政总支出的82.3%，年均增长15.8%。“十二五”期间，城镇居民人均可支配收入、农民人均纯收入年均分别增长13.02%和15.79%；2016年城、乡常住居民人均可支配收入分别达25595元、8855元，较上年增长8.3%、8.5%，增速分别居全省第1、第2位。

　　具体工作中，我们主要抓了以下几个方面：

　　一是坚持产业兴市，全力保持较快增长。纵深推进“农业倍增工程”，农业农村经济稳步发展。2016年，粮油生产实现“十三连丰”；与2010年相比，生猪出栏增长17%，茶叶产量增长157%，蔬菜产量增长39%，水果产量增长51%，水产品产量增长94.3%。市级以上农业产业化重点龙头企业达到213家，农民专业合作社达到3783个，家庭农场达到794个，产业化水平大幅提升。209个生产基地、342个产品通过无公害、绿色或有机认证，11个产品通过农产品地理标志登记保护，洋县创建为全国有机产品认证示范县。大力实施“工业翻番工程”，千方百计引导企业促销稳产保市场，工业经济始终保持平稳较快发展。2016年，规模以上工业增加值353.71亿元，是2010年的2.97倍；规模以上企业达到459户、比2010年增加89户，其中产值过亿元企业205户、比2010年增加149户；累计创建中国驰名商标6件、陕西名牌产品48个、省级著名商标207件和1个国家级、2个省级知名品牌示范区。强力推动“全域旅游工程”，旅游业与其他产业融合发展水平不断提升，带动了三产服务业加快发展。六年累计投入旅游发展资金129.2亿元，4A级景区从“十一五”末的1个增加到9个，星级饭店和商务酒店发展到423家。2016年，尤曼吉游乐水世界、诸葛古镇、汉山广场竣工投用；紫柏山创5A级景区通过省上资源评估，石门栈道、五龙洞成功创建4A级景区，新创建省级旅游示范县1个、特色名镇2个、乡村旅游示范村5个，汉中油菜花节跻身“全国十大花节花会”，连续3年开展旅游惠民活动，旅游总收入173亿元、是2010年的3.5倍。以天汉大道为主轴的百亿元核心商圈基本形成，连锁经营、电子商务等新型服务业快速发展。2016年社会消费品零售总额364.17亿元，是2010年的2.31倍。

　　二是坚持项目拉动，做大做强发展支撑。大力实施“千亿投资工程”，六年实施重点项目1304个，完成投资2110.78亿元。立体交通方面，建成汉中机场并开通6条航线、连通三大经济区8个城市，汉中火车站新站房竣工投用，十天高速汉中段、宝巴高速汉中至川陕界、西汉高速佛坪连接线、210国道西乡至镇巴段改造建成通车，西成高铁汉中段铺轨完成设计的65%，阳安二线、兰海高速青木川连接线等项目进展顺利，西乡至镇巴高速公路试验段开工建设，汉巴渝快速铁路列入国家交通规划。产业发展方面，实施工业技改项目228个，建成省级以上工业园区21个、现代农业园区37个、3A级以上景区22个，汉烟仓储及指挥中心、汉机精密高新技术产业基地、汉中茶城等项目建成投运，汉中褒河物流园、亿丰国际商贸城等项目进展良好，黄玉产业、石墨烯产业等项目取得突破。农田水利方面，建成高标准基本农田47万亩，补充耕地9.1万亩，保障建设用地10.4万亩；汉江综合整治建成堤防主体204公里，病险水库除险加固171座，云河水库、引汉济渭等工程取得重大进展。电力能源方面，大唐略电扩建和宁强巨亭等33座水电站项目建成投运，酒泉至湖南800千伏特高压工程汉中段、汉中330千伏主变扩建、机场110千伏变电站等项目完工；略阳至汉中第二输气管线基本建成，天然气管网达451公里、受益群众15万户。

　　三是坚持创新驱动，大力推动转型升级。汉中循环经济研究院设立全面启动。引进和招聘377名高层次人才，培育37家高新技术企业，开发50余种高新技术产品，大运飞机、数控机床、应变式传感器等在国内或国际处于领先水平；高新技术产业产值占规模以上工业产值的比重达21.4%；累计申请专利5626件、授权3556件，取得各类科技成果211项、获得省科技进步奖50项；攻克重大关键技术118项，其中国际领先12项、填补国内空白36项；科技进步综合水平居全省第4位。六年累计实施循环发展项目455个，初步形成4大循环产业体系、18条循环经济产业链条，中航工业集团与省政府共建汉中航空智慧新城进展良好，铺镇工业园创建为省级高新技术产业开发区，洋县现代材料园、城固五郎非金属产业园成为循环发展的新亮点。

　　四是坚持城镇带动，提升城乡发展水平。大力实施“城乡统筹工程”，加快推进以人为核心的新型城镇化。中心城区建设上，天汉大桥、龙岗大桥、滨江西路、江南西二环等一大批标志性工程建成投用，天汉文化公园文创街区开园迎客，汉江两岸滨水生态湿地公园基本建成，兴汉新区强势推进，打通了前进路西段、西新街东段等20余条“断头路”。2016年，城市建设重点年活动成效明显，82个重点项目完成投资309.35亿元，是上年的10倍。县城建设上，重点实施畅通、生态、点亮、文体、安居“五大工程”，整体提升承载能力和宜居水平。重点镇建设上，打造了一批个性鲜明、带动力强的特色名镇，青木川镇入选中国首批特色小镇，9个镇跻身省级重点示范镇或文化旅游名镇行列、占全省的14%。村庄社区建设上，重点打造的500个美丽乡村取得阶段性成效，建成新型社区、示范社区174个。全市城镇化率达47.8%、比2010年提高10.72个百分点，荣获“美丽中国·最美城镇”奖。

　　五是坚持文化名市，增强发展软实力。大力实施“文化振兴工程”，文化事业发展持续繁荣。先后建成市非遗保护及羌文化中心、群众大舞台等标志性工程，新建和提升“两馆一站”223个、重点镇综合文体中心16个、村（社区）文化活动室1906个、农家书屋2767个，广播、电视综合覆盖率分别达98.3%、99.1%；政府购买公共演出服务1000余场次，放映农村公益电影16万余场次；打造了《汉颂》、《青木川传奇》、《风过菜花黄》、《汉水汉中》、《汉源》等精品力作；省、市级文化产业示范基地达到24个，成功创建3个国家级文化先进县。海军“汉中舰”命名下水，汉中成为西北首个以地级市命名海军舰艇的城市。建成汉中体育运动中心和1495处体育惠民工程，“环中赛”汉中段比赛成功举办，全民健身活动蓬勃开展。

　　六是坚持生态立市，精心打造美丽汉中。严守发展和生态两条底线，持续推进“生态环境工程”。中心城区东排洪渠、燕子河治理等工程建成投用；六年累计实施减排项目222个，关停淘汰工艺落后企业或生产线126个；拆改燃煤锅炉960台，秸秆禁烧、清理城乡垃圾、淘汰“黄标车”、农村环境连片整治、扬尘治理成效明显。城市生活污水、垃圾无害化处理率分别达90%、99%。成功创建国家卫生城市和省级生态园林城市、环保模范城市，镇巴、宁强、佛坪建成国家级园林县城，省级园林县城实现全覆盖。六年累计完成造林153.75万亩，治理水土流失2343平方公里。

　　七是坚持改革开放，激发活力释放潜能。大力实施简政放权、放管结合、优化服务改革，市场活力加速释放；深入推进事业单位分类改革、镇村综合改革，服务管理水平得到极大提升；“一照一码”登记制度实现全覆盖，城市综合执法体制改革初见成效，农村土地确权登记颁证有序推进，户籍登记实现了城乡统一，国企改革稳步实施。扎实推进供给侧结构性改革，2016年，完成压减100万吨生铁、70万吨粗钢的去产能任务；商品房库存消化周期由上年的17个月下降为14.3个月，低于全省平均16个月的周期；规模以上工业企业每百元主营业务收入成本同比减少1.3元，降成本取得初步成效；短板领域投资加大，文化、体育、娱乐业投资增长83.2%，运输、仓储、邮政业投资增长81.4%，信息传输、软件和信息技术服务业投资增长65.7%。主动融入“一带一路”和长江经济带，深化与京津沪务实合作，打响“张骞故里文化经贸使团”品牌，招商引资到位资金六年累计2317亿元、年均增长15%，中交建、中电建、陕煤化、陕有色等世界或国内500强企业相继落户汉中。

　　八是坚持发展惠民，不断增进群众福祉。统筹推进“民生改善工程”、“扶贫攻坚工程”和“治理创新工程”，加快构建补缺兜底的社会保障体系。学前教育毛入学率超过全省平均水平，义务教育普及率进一步提升，高考上线率实现“五连升”，陕西理工学院升大成功，汉中职业教育集团挂牌运行。城乡一体的医疗服务体系基本建成，医疗卫生县域就诊率达80%。全面两孩政策稳步实施。城乡社会保障制度和政策得到全面落实。建成各类养老服务机构728个，每千名老人拥有养老床位数达到35张。移民搬迁15.28万户，建成各类保障性住房9.51万套，棚户区、土坯房改造分别完成6.96万户、3.66万户。住房公积金支持干部职工住房消费成效明显，资金使用率居全省前列。解决168万农村群众安全饮水问题，71.66万农村贫困人口实现脱贫，佛坪县可望在全省率先脱贫。深入开展双拥共建，荣获全国双拥模范城“三连冠”。安全生产、食品药品监管扎实有效。信访工作成效显著，网上信访工作经验在全国推广。矛盾纠纷化解、治安管理防范和打击违法犯罪力度加大，社会治安平稳可控，公众安全感调查结果居全省前列，荣获“全国见义勇为城市”奖，连续四年被评为全省综治及平安建设优秀市。

　　九是坚持依法行政，提高政府执行力公信力。健全完善政府议事规则，坚持政府领导班子集体学法和重大事项专家咨询制度，出台政府性债务、产业资金、国有资产（配置）等管理办法，政府科学民主决策水平得到较大提升。自觉接受市人大及其常委会的法律监督和市政协的民主监督，六年共邀请省、市人大代表、政协委员684人次列席政府重要会议，办理人大代表意见、建议和政协提案2987件、办复率100%。法治汉中建设迈出坚实步伐，荣获全国“六五”普法先进市。扎实开展群众路线教育实践活动、“三严三实”专题教育和“两学一做”学习教育，持之以恒落实“八项规定”精神，驰而不息纠“四风”、转作风。深入贯彻落实“三项机制”，严格落实“两个责任”，强化重点领域监督管理。2016年，全市“三公”经费较上年下降18.4%，“两代表一委员”对市政府工作满意度居全省第一。

　　与此同时，全市其他各项工作也取得了新的成绩。审计监督有力保障经济健康发展，供销系统扎根“三农”助力脱贫，人防体系平战结合能力明显提升，物价监管的市场机制渐成主导，民族宗教工作有序推进，地方志和档案管理工作持续加强，地震、气象防灾减灾能力不断增强，老龄、慈善、残疾人事业服务保障水平进一步提高，民兵和预备役建设较好发挥了服务发展、维护稳定的积极作用。

　　春华秋实，天道酬勤。过去的六年，我们始终坚持谋发展不停顿，自觉把汉中发展置于全国、全省大格局，深化市情认识，科学分析研判，开辟了“三市”建设新路径；始终坚持稳增长不松劲，牢记发展第一要务，千方百计保速度、扩增量、强实力，推动发展稳居全省第一方阵；始终坚持调结构不放手，着力转变发展方式，大力改善需求结构，持续优化产业结构，不断提高经济发展质量效益；始终坚持促改革不止步，大力破除体制机制障碍，全面释放发展活力动力；始终坚持保生态不自满，持续放大生态优势，蓄积转型发展新动能；始终坚持惠民生不懈怠，全力增进群众福祉，让全体人民共享发展成果。

　　奋斗充满艰辛，成绩来之不易。四届人大一次会议以来所取得的成绩，是在省委、省政府和市委的坚强领导下，市级各大班子精诚团结、聚力发展的结果，是全市各级党政组织顽强拼搏、开拓奋进的结果，是全市广大干部群众创新创造、苦干实干的结果。在此，我代表市人民政府，向奋战在各条战线上的工人、农民、知识分子、干部、驻汉部队指战员、武警官兵和公安干警致以崇高的敬意！向大力支持政府工作的各位代表、各位委员和同志们、朋友们表示衷心的感谢！

　　回顾过去六年工作，我们清醒地认识到，汉中发展还存在不少困难和问题：一是经济总量偏小，内生动力不足，经济持续向好的基础不牢，追赶超越的压力较大；二是产业层次偏低、结构不尽合理，创新驱动发展能力不强，资源环境约束趋紧，转型升级任务艰巨；三是群众收入不高，贫困人口量大面广，财政收入增速趋缓和民生刚性支出增加的矛盾较为突出；四是少数干部责任担当和进取意识不强，不作为、不会为、不善为的问题依然存在，法治政府建设和干部作风建设仍是长期任务。对于这些问题，我们必须高度重视，切实加以解决。

　　二、今后五年的主要目标和任务

　　今后五年是我市奋力追赶超越、同步全面小康、更高水平建设“三市”的决胜阶段。全市经济社会发展的总体思路是：全面贯彻习近平总书记系列重要讲话精神和中省战略决策，统筹推进“五位一体”总体布局和协调推进“四个全面”战略布局，切实按照市五次党代会的安排部署，牢固树立和贯彻落实新发展理念，适应、把握并引领经济发展新常态，以提高发展质量和效益为中心，以推进供给侧结构性改革为主线，以“三项机制”为保障，围绕追赶超越、绿色循环、转型升级，加快实施“六大战略”，发展壮大“六大产业”，强力推进“十大工程”，建好“五大园区（村）”，构筑“三大项目支撑体系”，做优唱响“两汉三国·真美汉中”城市品牌，全力打赢精准脱贫攻坚战，建成区域中心城市、文化旅游强市、陕西最美城市。

　　主要奋斗目标是：五年期间，经济增速高于全省平均水平2个百分点，生产总值年均增长10%以上，到2021年达到2000亿元；全社会固定资产投资年均增长20%、到2021年达到3000亿元；规模以上工业增加值年均增长12%以上、到2021年达到660亿元；城乡常住居民人均可支配收入年均分别增长11%、12%，到2021年达到44189元、16105元，以上指标增速均超过全省平均水平。战略性新兴产业占生产总值比重达16%，常住人口城镇化率60%，旅游总收入年均增速15%以上，以上指标均达到全省平均水平。科技进步贡献率58%，文化产业增加值占生产总值比重达3.5%，非公经济增加值占生产总值比重达53%，以上指标均接近全省平均水平。到2021年，生产总值、财政总收入等指标接近我省的渭南市和毗邻的达州市。经过五年努力，经济总量继续领跑陕南三市，在全省位次前移，在陕甘川毗邻地区优势更加明显。重点抓五个方面：

　　━━全力推动产业升级。深入实施《中国制造2025汉中实施方案》，推动装备制造、现代材料、绿色食药、文化旅游、养老养生等产业向高精尖、集约化迈进，集中力量打造12条循环经济产业链和6大循环农业产业链，加快形成特色鲜明、多点支撑、集群发展的现代产业体系。到2021年，一产占比调整到10%左右、三产占比达到40%以上，工业园区产值突破2000亿元，实现旅游总收入360亿元、比2016年翻一番；建成国家高端装备制造业循环示范基地和绿色有机农产品基地，成功创建国家全域旅游示范市。

　　━━全力完善基础设施。建成西成高铁、阳安二线，开工建设汉巴渝快速铁路汉中段；建成西乡至镇巴高速公路，实现县县通高速、平川县城通一级路、重点镇及4A级景区通等级公路，全市公路总里程突破2万公里；拓展汉中城固机场“四向融入”新航线，建设洋县、宁强等通用机场，梯次推进秦巴深山公路环线建设，基本形成“四环红太阳”的立体交通网。开工建设750千伏电网工程，建成330千伏南郑、西乡等变电站，形成汉中电网与陕西主网双回通道，建成覆盖全市高速公路服务区、干线公路的电动汽车充电网络，中心城区建成便捷化电动汽车充电系统。实现“气化汉中”三级全覆盖。开工建设城固焦岩水库及12个骨干水源工程，大力实施汉江生态修复治理、引汉济渭等工程。到2021年，重大项目累计完成投资4000亿元，带动全社会投资累计达到1万亿元。

　　━━全力加快城镇化步伐。扎实推进以人为核心的新型城镇化，构建以中心城市为核心、县城为节点、特色名镇为依托、美丽乡村为点缀的“一心一带三辐射”城镇群。在中心城市建设上，深入实施“双百”战略，加快打造“一江两岸”、滨江新区、兴汉新区、龙岗新区，大力推进棚户区、城中村改造，深入实施城市道路、地下综合管廊、雨污分流、生态修复等工程，全面建设“海绵城市”、“智慧城市”，城市宜居度显著提升。在县级城市建设上，依托资源禀赋，彰显山水园林、生态宜居特色，增强综合承载能力；加快南郑、城固撤县设区和勉县、西乡撤县设市步伐。在镇村建设上，加快推进城乡公共服务均等化，实现城乡机会均等、共建共享。

　　━━全力增进群众福祉。坚决打赢脱贫攻坚战，到2018年，现行标准下的50.23万贫困人口全部脱贫，贫困村全部退出，贫困县全部摘帽。主要就业指标稳居全省前列。实施居民收入增进计划，城乡居民收入增速高于全省平均水平1至2个百分点。着眼建设区域中心城市、文化旅游强市的需要和发展养老养生等产业的巨大需求，高标准谋划和推进社会事业发展。推行13年免费教育，全面普及学前三年教育，启动汉中教育现代化行动计划，到2021年教育发展水平在全省和毗邻地市处于领先位次；深入实施“健康中国2030规划纲要”，建立覆盖城乡居民的基本医疗卫生制度；培育壮大文化产业，不断健全公共文化服务体系；完善社会保障体系，实现基本制度定型完备、法定人群全面覆盖、保障标准合理调升目标；健全住房保障体系，全市城镇人均住房面积达到46平方米；大力发展养老事业，每千名老人拥有养老床位数达到40张以上。

　　━━全力放大生态优势。推进大气、水体、土壤污染防治，狠抓城乡环境综合整治，确保环境质量稳居全省前列。严守耕地保护红线，大力推动资源能源高效利用，积极推广清洁能源，着力形成绿色低碳循环生产生活方式。实行最严格的水资源保护制度，加快实施山水林田湖一体治理，继续推进水源涵养林建设，强化汉江、嘉陵江流域生态系统保护与修复，筑牢生态安全屏障。到2021年，森林覆盖率达到63%，单位生产总值能耗下降15%，建成国家森林城市、环保模范城市和生态文明示范市。

　　实现上述目标任务，我们必须坚持做到以下几个方面：

　　一要勇于担当追赶超越的历史使命。追赶超越是引领汉中今后五年发展的最强音和主基调。经过近年来的发展，汉中已经站在新的历史起点上，但仍滞后于全国、全省“四化同步”进程，在全省和毗邻地区竞相赶超的发展格局中，面临着前有标兵、后有追兵的紧迫形势。只有赶超，才有出路。我们要切实担负起推进汉中追赶超越的历史使命，突破盆地意识的桎梏，站在秦岭、巴山之巅谋划汉中发展，在拼抢机遇中主动作为，在克难攻坚中奋起直追，在争先创优中大胆超越，奋力谱写陕西追赶超越的汉中篇章。

　　二要牢牢把握经济新常态的本质要求。始终坚守经济新常态这个大逻辑，把转方式调结构放在更加突出的位置，坚持传统产业升级改造和新兴产业培育壮大并举，加快淘汰落后生产技术、工艺、装备，推动发展朝着产业结构更高级、经济结构更合理、质量效益更好的阶段迈进。更加重视市场配置资源的决定性作用，使经济发展更多依靠科学技术、体制机制、商业模式的全方位创新来推动，更多依靠人力资本、知识资本、技术资本的优化和提升来支撑，加快培育形成新的增长动力。

　　三要始终遵循系统化思维的工作方法。坚持把系统论的思想方法作为搞好工作的基本要求，强化“四化同步”理念，推动信息化和工业化深度融合、工业化和城镇化良性互动、城镇化和农业现代化相互协调。强化以人为本理念，坚守以人民为中心的发展思想，始终把人民利益放在首位，真心实意为群众办实事、做好事，不断增进人民群众获得感。强化改革创新理念，直面经济发展新常态下面临的矛盾和挑战，抓关键、善统筹，用改革破解难题，靠创新激发活力，全面释放改革红利。

　　四要发挥三大项目支撑体系的带动作用。全面推进“十大工程”建设，使之成为推动经济社会发展、实现追赶超越的全面性布局、骨干性项目支撑体系；倾力打造“一环、两城、三园、四极”十大重大项目，使之成为实现追赶超越的战略性布局、突破性项目支撑体系；分年度谋划实施若干产业类、基础设施类、环保类、文化事业类、民生类等大项目或实事好事，使之成为实现追赶超越的群体性布局、基础性项目支撑体系，多点发力、多元支撑，奋力实现追赶超越。

　　我们坚信，经过全市上下的不懈努力，一个充满生机与活力的新汉中，将会加速崛起在秦巴大地，成为陕西追赶超越的重要一极。

　　三、2017年工作重点

　　今年是全面贯彻市五次党代会精神的开局之年，也是全面建设区域中心城市、文化旅游强市、陕西最美城市的起步之年。全市经济社会发展的预期目标是：生产总值增长10%左右，全社会固定资产投资增长20%，规模以上工业增加值增长11%左右，社会消费品零售总额增长12%，财政总收入同口径增长10%、其中地方财政收入同口径增长7.6%，城乡常住居民人均可支配收入分别增长9%和10%，单位生产总值能耗下降3.2%，人口自然增长率控制在6‰以内，居民消费价格指数控制在3%左右，城镇登记失业率控制在3.9%以内，化学需氧量、氨氮、二氧化硫、氮氧化物、挥发性有机物减排任务全面完成。围绕上述目标，重点抓好六个方面：

　　（一）全力稳增长扩投资，迈出追赶超越新步伐

　　毫不动摇地把稳增长扩投资作为首要任务，想尽千方百计，调动一切力量，大力振兴实体经济，加快培育“四上企业”，推进经济保持较快增长。

　　力促工业持续向好。围绕新一轮“工业翻番工程”，大力落实省、市工业稳增长促投资的政策措施，市财政拿出资金以奖代补，用于规上企业培育、骨干企业扩销、企业技术改造、省级园区创建等方面，激励企业提速增效；加快建设陕飞运X飞机生产及总装扩能配套、燕航精工年产10万台汽车自动变速器生产线等一批重点技改项目，着力培育新的增长点；引导和带动企业走“名、特、专、新、精”发展之路，打造一批“小巨人企业”、“瞪羚企业”和“规上企业”，全年培育中小微企业2000户、中小企业梯队企业100户、科技型企业50户、瞪羚企业30户、规模以上企业30户、亿元企业10户；强化政银企合作，扩大范围、提升层次，充分发挥财政资金的杠杆作用，撬动金融资本投身实体经济发展。

　　着力扩大有效投资。坚持不懈实施“千亿投资工程”，强力推进六大板块304个重点项目，完成年度投资654亿元，确保西成高铁、宝汉高速汉中以北段等29个收尾项目尽早完工，阳安二线、西镇高速等98个续建项目加快推进，天汉大剧院、244国道汉台至南郑一级公路等74个计划新开工项目如期启动，秦巴深山公路环线、民用货机研制等103个前期项目取得重大突破；密切跟踪天然气、石墨烯、黄玉、天坑群地质遗迹等资源勘探进展，突出基础设施提升、产业集群建设、现代服务业发展和民生保障、脱贫攻坚、生态环保等，策划一批稳增长、增后劲、利长远的重大项目；加大中省项目资金和国家专项资金争跑力度，全面推广PPP模式，认真落实好征地、拆迁补偿安置政策，加大工程建设领域环境整治力度，确保项目建设顺利实施，引领全社会固定资产投资完成1450亿元。

　　加快发展现代农业。以农业供给侧结构性改革为抓手，以“十大绿色农产品基地”建设为引领，持续推进“农业倍增工程”，粮、油产量分别稳定在100万吨、17万吨，出栏生猪410万头，生产蔬菜220万吨、水果40万吨、茶叶3.9万吨、水产品4.2万吨，林下经济产值16亿元。大力发展订单农业，持续发展示范家庭农场、专业合作示范社，扩大新型职业农民队伍。加强“三品一标”认证管理，推进质量安全追溯体系建设。深化与京、津、沪等地区的农业经贸往来和务实合作，扩大优质农产品的市场占有率。扎实做好农业普查工作。

　　持续推进全域旅游。着眼高铁时代到来，围绕打造知名旅游目的地城市，更加注重游客的感悟和需求，加快实施“全域旅游工程”，加大国家全域旅游示范市创建力度，建设重点文旅项目30个、完成年度投资195亿元，全力加快紫柏山、华阳、黎坪、青木川景区创5A步伐。精心谋划和推动“汉中天坑”地质遗迹旅游开发和“大汉中、古栈道”文化旅游品牌建设，积极开发休闲度假、养老养生、康体健身、乡村旅游等新业态。加大竹园五星级酒店、龙岗温泉酒店和农家宾馆、自驾车营地建设力度，建成汉中旅游集散咨询服务中心、市级智慧旅游数据中心，加快智慧旅游在线服务平台建设。大力开展“放心消费”创建工作，让放心消费、舒心消费成为汉中旅游消费体验新名片。深化“三建设一整治”，创新举办油菜花节等品牌推介活动，狠抓旅游形象宣传。全年接待游客3850万人次、增长18%，旅游总收入力争达到210亿元、增长21%。

　　大力发展现代服务业。开工建设汉府长乐坊、华汉新世纪等商业综合体，加快亿丰国际商贸城、普汇中金世界港、社区便民消费工程、农村商品和农产品流通网络等项目建设，全年实施农村市场建设项目17个。扎实开展电子商务示范创建活动，加大电商、物流龙头企业引进力度，培育电子商务应用企业50户，新建电商扶贫网店318个。坚持生产性与生活性服务业并举，对接六大产业，大力发展研发设计、物流仓储、金融服务等生产性服务业；着眼群众需求，大力培育订制类消费、大众化餐饮等消费热点，千方百计扩大市场消费。全年社会消费品零售总额超过400亿元。

　　（二）久久为功调结构，培育追赶超越新支撑

　　更加注重把追赶超越体现在发展质量上，加快经济结构战略性调整，不断提升发展的整体素质、综合效益和核心竞争力。

　　强化园区集约承载。按照要素集聚、产业集群、服务集成的要求和资源共享、区域共建的思路，加快完善各类园区基础设施，大力推进市、县互动的服务平台建设，更好地服务市场主体。积极探索以企业为主体的园区开发模式，组建和引进具有独立法人资格的基础开发公司进行市场运作、综合开发，建立健全以资金融通、信息服务、人才培训为主的园区配套服务体系。支持航空智慧新城、创智产业园、铺镇工业园等园区围绕功能定位，开展产业链招商，促进大企业、大项目向园区集聚，以特色产业园区构建特色产业集群。

　　做优产业绿色循环。以汉江循环经济产业带为核心，重点打造4大循环体系、18条循环产业链，形成协作配套、耦合共生的循环产业体系。推动传统制造业绿色化改造，大力发展节能环保、新能源等绿色产业，拉动绿色消费，推动转型升级。实施全民节能行动计划，推进工业、建筑、交通、公共机构等重点领域节能减排，加强高耗能行业能耗管控，大幅降低资源消耗强度。

　　释放科技创新潜能。实施创新驱动战略，加大国家级创新型试点城市创建力度，建成陕西省科技资源统筹中心汉中分中心，启动汉中高新区科技资源统筹中心建设工作，吸引科技型企业来汉开展研发、中试和落地项目。加快构建以企业为主体的创新体系，重点培育高新技术企业30家，支持企业加大研发投入，在高端装备制造、生物医药等领域突破一批关键技术。深入推进军民融合，壮大民用产品制造产业，打造国家级军民融合发展示范基地。加强与大专院校、科研院所的合作，加大各类人才的培养引进力度，利用创业苗圃、创客空间、创业公寓和创业交流等孵化平台，推动大众创业、万众创新。

　　打造新兴产业亮点。运用互联网、云计算、大数据等新一代信息技术，加快“数字汉中”建设。支持陕飞公司做大规模，构建航空制造产业集群，培育壮大高端装备制造产业。依托资源优势，选择有潜力的企业精准招商，促进现代材料产业突破发展。大力发展生物提取、生物制造、生物医药等产业，积极开发植物制剂、保健食品、膳食补充剂等高端产品。推动文化创意和创新设计在各领域应用，培育更多新产品、新服务以及多向交互融合的新业态。

　　（三）紧锣密鼓促改革，释放追赶超越新动能

　　改革开放永无止境。面对实现追赶超越、建成全面小康的艰巨任务，唯有改革才有出路，唯有开放才有未来。

　　持续深化“三去一降一补”。大力推动钢铁企业资产重组，完成煤炭“去产能”任务；继续抓好“三房打通”政策落实，提高棚户区改造货币化安置率，全市商品房库存减少3%；大力推进金融创新和投融资渠道拓展，组建汉中金融控股投资集团，扩大与上市公司的合作，加强与资本市场对接，抓好投融资平台和体系建设，支持企业在主板、新三板上市，缓解产业发展、基础设施建设和民生改善的资金困难。加大闲置土地处置力度，提高土地集约节约利用水平，促进要素逆向流动常态化、规范化；认真落实省市关于降成本的政策措施，因地制宜搭建汉中企业低成本运营平台，进一步减轻企业成本负担，进而支持新项目落地和招引；全面落实省上十大补短板举措，提升发展的协调性、均衡性和可持续性。

　　全面开展农业供给侧改革。认真贯彻中央1号文件精神，推进农村一二三产业融合，优化种养殖、深加工和流通服务业布局，构建“接二连三”的全产业链。推广水肥一体化、生物防治等绿色生产技术，加强农业废弃物无害化处理，实现资源利用高效、产地环境良好、产品质量安全。落实好承包土地三权分置政策，统筹推进农村土地征收、集体性建设用地入市、宅基地改革等试点，加快南郑县农村土地承包经营权抵押权贷款国家试点进程。

　　稳步推进行政体制改革。以更有力举措推进“放管服”改革，再取消下放一批含金量高的审批事项。全面推行工商注册全程电子化，落实“多证合一”登记制度，推进“双随机一公开”。健全政府数据共享开放体制机制，进一步强化事中事后监管。稳步推动行业协会、商会脱钩试点，完成国有林场改革、城市执法体制改革。完善政企服务对接机制，把促进民营经济发展纳入目标责任考核。

　　强推基础性关键性改革。按照中省国企改革的要求，抓好略钢、收获机械厂、粮油总公司改制，启动一批条件成熟企业的改制工作，推进国有企业“三供一业”移交、剥离办社会职能。立足改革和发展，做强、做大、做优国有企业，完善并发挥好汉中国企在承担国计民生保障、重点产业引导和战略新兴产业引领方面的作用。进一步完善现代产权制度，深化不动产登记制度改革，推进产权保护法制化，加强对各种所有制组织和自然人财产权的保护。

　　全力以赴扩大对外开放。围绕打造“丝路源点城市”，强化“四向融入”举措，扩大对外开放步伐。加快出台支持“汉商”回归创业的优惠政策，弘扬企业家精神，壮大企业家队伍，激励汉中籍在外客商回报桑梓。要围绕六大产业发展，超前谋划、科学策划包装重大项目，深化与央企、省属企业的合作，积极参加中、省举办的系列投资促进活动，继续开展“张骞故里文化经贸使团”系列招商活动，瞄准国际国内500强、创新行业100强等企业招大引强，全年引资到位560亿元、增长12%。稳步推进汉中口岸、汉中海关、电子口岸和保税仓库建设。

　　（四）趁势而上抓脱贫，增添追赶超越新活力

　　全面落实“六个精准”、“五个一批”举措，持续推进“脱贫攻坚工程”，确保426个贫困村、18.86万贫困人口脱贫退出，汉台区整体脱贫、留坝县脱贫摘帽。

　　大力完善基础设施。实施特困户“交钥匙”工程，加快农村危旧土坯房改造步伐，完成建档立卡易地扶贫搬迁建房2.58万户7.05万人，生态避灾移民搬迁8000户。持续加大水、电、路等基础设施建设力度，完成剩余26个贫困村通村水泥路建设任务，解决3.87万贫困人口饮水安全问题。加快标准化卫生室、文化书屋、健身设施、养老服务机构建设，大力推进贫困镇义务教育学校和贫困群众医疗、养老保险全覆盖，为增收脱贫创造良好条件。

　　多措并举促进增收。注重政策叠加，推动扶贫与现代农业、全域旅游、电子商务等相互融合，扎实开展“十百千万”产业扶贫工程，实现产业扶贫全覆盖。坚持生态补偿促增收，挖掘护林员岗位开发潜力，扩大符合条件贫困群众的工资性收入。加大教育扶助和技能培训力度，扩大汉中职业技术学院对贫困户子女的免费教育，增强转移就业、增收致富能力，达到“培训一人、脱贫一户”的效果。落实好低保和符合规定建档立卡贫困人口“两线合一”工作，夯实农合、大病保险、医疗救助三道防线，对特殊贫困群体实现优惠政策全覆盖。

　　各方联动共同帮扶。建立脱贫攻坚年度“任务清单”和“责任清单”，实行政策、项目、资金、人才“四倾斜”，市县财政按不低于一般性预算收入2%的比例安排专项扶贫资金，确保增幅不低于20%；全年新增小额扶贫贷款10亿元；新建贫困村互助资金协会100个。积极推进中央单位定点帮扶、省级单位“两联一包”、市县部门单位和干部包村扶持、千企千村扶助行动和“苏陕对口协作”。驻村帮扶群众满意度达到90%以上，贫困人口识别退出精准度达到95%。

　　（五）坚定不移保生态，厚植追赶超越新优势

　　把生态建设贯穿于经济发展的全过程和建设陕西最美城市的各方面，加快建设天更蓝、地更绿、水更清、环境更优的美丽汉中。

　　持续整治环境污染。推进垃圾焚烧发电厂、铺镇污水处理厂等五大治污项目，强化“六厂（场）一车”等重点企业脱硫、脱硝设施的运行监管，确保减排任务圆满完成。深入推进土壤污染防治，划定生态保护红线，精准落实治污降霾六项措施，空气优良天数、PM2.5、PM10年均浓度达到考核要求；全面推进“河长制”，统筹抓好农村环境综合整治和面源污染防治，加快西汉水嘉陵江锑污染、废弃裸露矿山等生态恢复治理，汉江、嘉陵江出境水质稳定达到二类标准，城市集中式饮用水水源地水质达标率98%、农村饮用水水源地无重大污染。持续开展环保“零点行动”，全面整改中、省环保督察发现问题。

　　大力开展生态建设。深入实施天然林保护、退耕还林等重点生态项目，持续推进经济林建设和退化防护林改造，构筑绿色生态屏障；坚持见空布绿、见缝插绿、拆墙透绿、拆违补绿、生态增绿，提升城市绿化水平。全年完成造林20万亩。做好冷水河入汉江河口整治与生态综合治理项目前期工作，全面铺开水保重点项目建设，治理水土流失500平方公里。逐步扩大“旱改水”试点，巩固“双退”成果，完成1万亩改地任务。

　　建设宜居宜业家园。启动新一轮城市总体规划编制和多规合一工作，优化城镇空间结构布局，加快打造沿江串珠式城镇带、汉中盆地城镇群。中心城区建设上，大力开展城市建设深化年活动，实施重点项目412个、完成投资338亿元，全力推进滨江新区、龙岗新区开发和东关、南关等十大棚改街区改造，开工建设汉宁路、望江路等“断头路”改造项目；兴汉新区要加快推进梁中路、轩辕新桥、汉源湖等项目，开工建设西华路、汉乐府等项目。深入开展“五城联创”，综合整治交通拥堵、渣土运输、占道经营、物业管理等突出问题，确保国卫复审顺利达标、国园创建一举成功、国森和国模创建全面启动、国文创建基础扎实。县城建设上，紧扣发展定位，依托资源禀赋，突出抓好各类基础设施建设，完善服务功能，提升承载能力。特色名镇建设上，全力推进39个省、市重点镇、特色专业镇建设，引领和带动全市小城镇快速发展。社区和村庄建设上，建好48个农村社区服务中心、2个街道社区服务中心、22个社区服务站、4个社区室外活动广场；加快打造10条美丽乡村风景线和20个旅游精品村，新启动建设100个美丽乡村示范村，培育800个清洁乡村、300个生态乡村、200个幸福乡村、20个魅力乡村。

　　（六）以人为本惠民生，分享追赶超越新成果

　　按照守住底线、突出重点、完善制度、引导舆论的思路，深入细致地做好社会托底工作，不断扩大人民群众获得感。

　　持续完善社保体系。认真落实就业创业10项优惠政策，全年城镇新增就业3.2万人，转移农村富余劳动力75万人次以上。加快落实全民参保登记计划，不断扩大社会保险覆盖范围，“五大保险”参保率超过98%。加大城乡低保和临时救助、医疗救助力度，完善对困难职工、孤残群体、农村留守人员和妇女儿童关爱服务体系。推动养老服务产业加快发展，着力形成机构养老、社区照料、农村互助幸福院、居家服务“四位一体”的养老服务格局。全年新建和改扩建各类养老服务机构125个，每千名老人养老床位达到37张。继续提高住房公积金使用率，建成全省一流的信息管理系统。持续深化保障房建设，完成棚户区改造1.2万户。

　　协调发展社会事业。推进各类教育均衡发展，重点实施170所“全面改薄”及县城以上城区中小学校建设项目，完成11所公办幼儿园新建、168所学校幼儿园标准化创建任务，建成3个国家义务教育发展基本均衡县、3个省“双高双普”县区。以群众满意为出发点，深化城市公立医院改革、取消药品加成，逐步完善分级诊疗制度，实行公立医院药品采购“两票制”，推进二级以上医院对口支援镇卫生院等制度，提升公共卫生服务水平。稳妥实施全面两孩政策，深化计划生育服务管理改革，完善配套保障措施，全年人口自然增长率控制在6‰以内。扎实开展全民健身活动，继续推进体育惠民工程，建成汉中水上运动中心和滨江体育公园，办好市第六届运动会，精心做好第十四届全运会汉中比赛项目的筹备工作。社会事业事关民生大计，需加大审计监管力度，及时发现问题，严惩违规违纪违法。

　　全力推进文化振兴。持续加强公共文化服务体系建设，深入挖掘历史文化资源，以陕南民歌、汉调桄桄等地方特色文化品牌和优势文艺门类为载体，精心打造一批富有汉中特色、在全省全国有影响的大戏好书名作，力争在省第八届艺术节创造佳绩。加快工人文化宫、青少年宫、妇女儿童中心、老年服务中心、军休干部活动中心、广播电视台融媒体中心等市级文化设施和县区文化馆、图书馆、基层综合性文化站建设，组织文化惠民演出600场，放映农村公益电影2万场次。加快藏羌彝文化产业走廊等重大项目和龙岗遗址文化生态园等产业园区建设，推动文化产业整体提升。

　　切实维护安全稳定。巩固“三查三打三整改”活动成果，切实维护社会大局和谐稳定。加强社会组织的培养和监督管理，更好发挥其在公共事务管理中的作用。加强基层防灾体系和应急处置能力建设，增强人民群众安全感。持续开展风险隐患排查整治活动，依法推进打非治违，坚决防范和遏制较大以上安全生产事故。全面加强行政监管、稽查执法、技术支撑体系建设，建成市级食品药品检验检测中心和4个县级区域分中心，至少建成1个省级食品安全示范县区；扎实开展高频次、全覆盖的检查抽查，着力防范和化解系统性食品药品安全风险，确保“舌尖上的安全”。坚持“谁执法、谁普法”，深入推进“七五”普法，突出抓好法律援助、社区矫正；继续加大领导干部“四访”和包案化解工作力度，坚持依法治访，严厉打击违法上访行为和无理“缠访闹访”人员，维护正常的信访秩序。创新完善治安防控体系，依法开展严打整治，强化公共安全监管，努力建设更高水平的平安汉中。

　　四、建设人民更加满意政府

　　民之所望，施政所向。肩负着追赶超越的历史使命，我们必须以“激情、干净、超越”主题作风建设为契机，全面提升执行力、公信力、创新力，努力建设人民更加满意的政府。

　　（一）强化“四个意识”，始终做到政治过硬、信念坚定。强化政治意识，坚定理想信念，保持政治定力，坚守清正之风，自觉践行“三严三实”，坚决做到无私无畏、绝对忠诚。强化大局意识，主动适应新常态、顺应新形势、落实新要求，紧紧围绕中省和市委决策部署，发扬“踏石留印、抓铁有痕”的韧劲，推进各项工作高效落实。强化核心意识，严守政治纪律、政治规矩，始终与以习近平总书记为核心的党中央保持高度一致，自觉做到“四个服从”。强化看齐意识，经常主动地向中央看齐，向习近平总书记看齐，向中省决策部署看齐，高标准、严要求，推进又好又快发展。

　　（二）落实“三项机制”，始终做到求真务实、敢于担当。增强执政为民的担当意识，勇于打破思维定式，敢于破除固有藩篱，着力破解招商引资、项目融资、机制创新等方面难题。强化对为官不为的追责力度，下功夫治理廉而不为、廉而不勤、懒政怠政等突出问题，层层传导压力，逐级夯实责任，激励政府系统干部职工主动担当、积极作为。加强对为官有为的支持奖励，支持改革、鼓励创新、宽容失误，真正让敢担当、善创新、有作为的干部没顾虑、有舞台，推动工作提速、效能提高、形象提升，凝聚干事创业、争创一流的强大合力。

　　（三）遵循“两部法规”，始终做到纪律严明、廉洁清正。严守《党内政治生活若干准则》和《党内监督条例》，坚持执纪在前，厉行“八项规定”精神，推动“两个责任”落地生根。强化行政监察和审计监督，完善政府采购、公共资源管理、招投标监管等机制，严格落实公务接待、办公用房、公务用车等制度，进一步降低行政成本。从严廉洁治政，健全惩治和预防腐败体系，坚持有腐必反、有贪必肃，从严查处以权谋私、失职渎职和损害群众利益等腐败问题，努力做到干部清正、政府清廉、政治清明。

　　（四）坚守“一条主线”，始终做到规范用权、服务发展。认真落实全面依法治国战略，严格按照法定程序、法定权限、法定职责行使权力，深入推进政府职能法定化和事权规范化，全面落实权力清单、责任清单、负面清单等制度，做到权力事项清、流程清、责任清。自觉接受市人大法律监督、市政协民主监督和社会各界监督，广泛听取民主党派、工商联、各人民团体及无党派人士的意见和建议，完善议事规则，不断提高政府科学化、民主化决策水平。始终牢记为民宗旨，切实把改革发展成果体现在人民生活水平的提高上，让全市人民过上更有尊严、更为富裕、更加幸福的生活。

　　各位代表！追赶超越千帆竞，全面小康使命艰。让我们在中共汉中市委的坚强领导下，不忘初心，继续前进，循环发展、追赶超越，以更加坚定的信心、更加昂扬的斗志、更加务实的作风，全面开启“三市”建设新征程，以优异成绩迎接党的十九大和省十三次党代会胜利召开！
